# Nasal probe and toothpick tool use by a wild female bearded capuchin (*Sapajus libidinosus*)

**DOI:** 10.1007/s10329-015-0470-6

**Published:** 2015-04-12

**Authors:** Michael Haslam, Tiago Falótico

**Affiliations:** RLAHA, School of Archaeology, University of Oxford, Dyson Perrins Building, South Parks Road, Oxford, OX1 3QY UK

**Keywords:** Capuchin monkey, *Sapajus libidinosus*, Stick tool, Nasal probe, Toothpick

## Abstract

We report the first observation of probe tool use by a wild adult female bearded capuchin (*Sapajus libidniosus*), at Serra da Capivara National Park (SCNP), Brazil. This individual used several stick tools and one grass stem to probe her nostrils, usually triggering a sneeze reaction, and also used stick tools to probe her teeth or gum. Both of these behaviours were accompanied by inspection and licking of the tool following use. We have termed these self-directed actions nasal probe and toothpick, and neither has been previously reported in wild capuchins. While stick tool use is common among foraging male capuchins at SCNP, the novel and at present idiosyncratic activities performed by the female monkey add to the known behavioural repertoire for this species.

**Electronic supplementary material:**

The online version of this article (doi:10.1007/s10329-015-0470-6) contains supplementary material, which is available to authorized users.

## Introduction

In the past decade, the establishment of long-term field studies of wild Brazilian bearded capuchin monkeys (*Sapajus libidinosus*) has revealed abundant evidence of tool use in this species (Ottoni and Izar 2008). Most attention has been given to capuchin use of stones to break open encased fruits and seeds, tasks for which they select and transport suitable tools to nut-cracking sites, leaving distinct traces of this activity on the landscape (Visalberghi et al. 2013; Haslam et al. 2014). However, wild capuchin groups in Serra da Capivara National Park (SCNP) demonstrate a wider range of tool materials and behaviour than seen elsewhere, including use of stones in sexual displays and digging tasks, and use of vegetative probes to extract small prey from crevices, threaten dangerous animals, and obtain foods such as honey (Mannu and Ottoni 2009; Falótico and Ottoni 2013, 2014).

Here, we report two new self-directed plant tool behaviours performed by a female *S. libidinosus* at SCNP, the first evidence of adult female probe tool use in wild capuchins. Intriguingly, use of probe tools is almost exclusively practiced by male capuchins at SCNP, comprising 97 % of observations during a long-term study of SCNP capuchin tool-use behaviour (Falótico 2011; Falótico and Ottoni 2014). No adult female has been seen to use a probe tool for foraging, and the only prior instance of an adult female using a stick tool was when one capuchin used a stick to poke the individual she was grooming (Falótico and Ottoni 2014). There is no ecological explanation for the stark sexual bias in probe tool behaviour at SCNP, since females prey on and consume the same plant and animal items to which males direct their probe tool use. At present, a bias towards male capuchins at SCNP, in either learning opportunities or ontogeny, are being considered as possible explanations.

## Methods

SCNP is located in Piauí, northeastern Brazil. The climate is semi-arid *Caatinga*, with a mosaic of xerophytic vegetation and patches of deciduous forest within narrow valleys (de Araújo et al. 1998). The current study was conducted in September 2014 with the Jurubeba group, which has been studied since 2004 (Mannu and Ottoni 2009) and consisted of 54 identified individuals at that time. The reported observations occurred at 8°52.5′S, 42°38.0′W, and were conducted opportunistically by video recording with a Sony PJ530E camcorder.

## Results

**Fig. 1:**
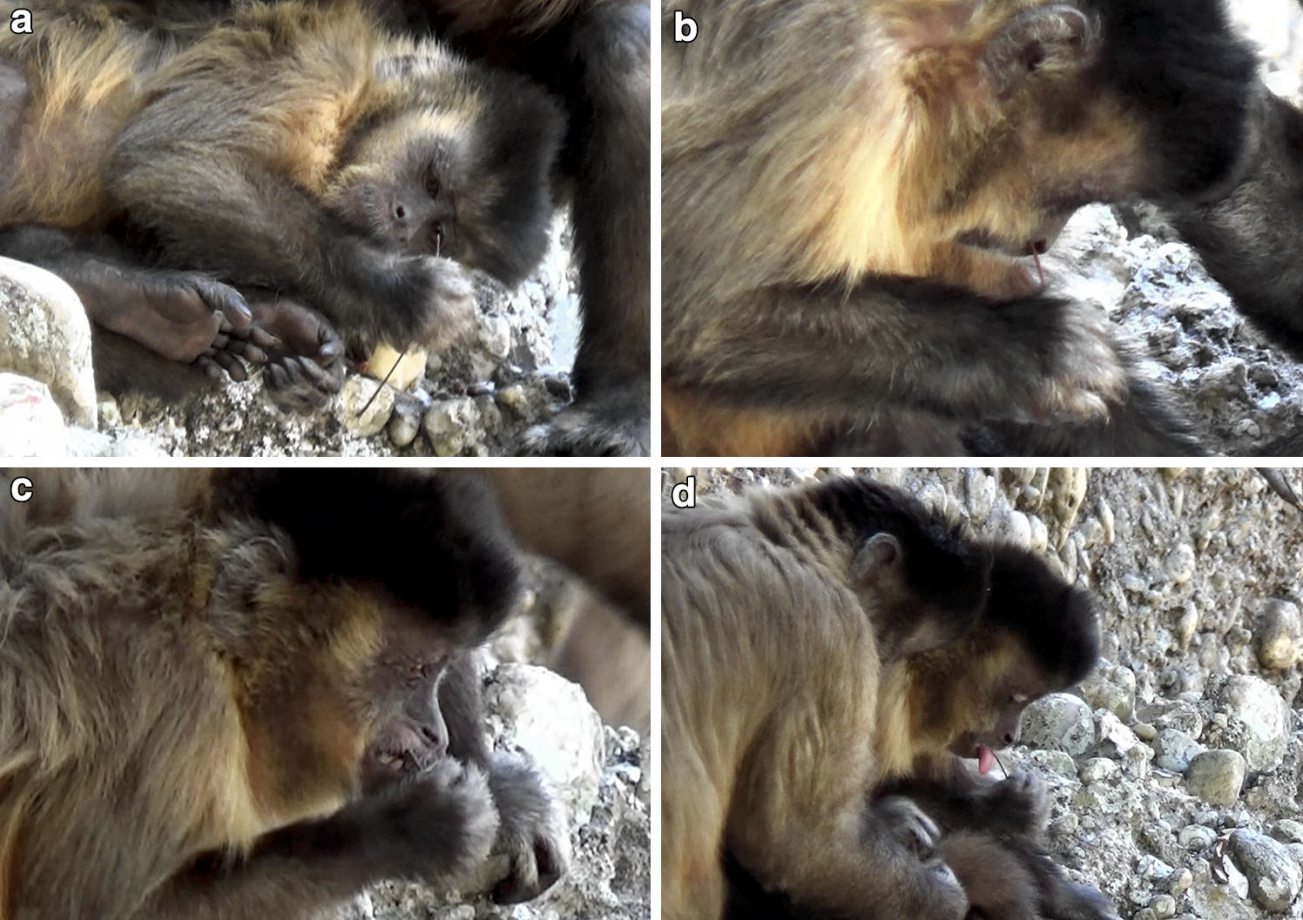
Adult female bearded capuchin probe tool use, Serra da Capivara National Park: **a** inspection of stick tool; **b** nasal probe; **c** toothpick; **d** licking the tool following toothpick behaviour. The tool in the pictures is Tool B

On 28 September 2014, at approximately 10:14 am, MH observed one adult female (Acácia) being groomed by an adult male (Choquito). While being groomed, Acácia was seen using at least four tools to probe her nose and mouth over a period of about 5 min (Online Resource 1). The first observed probe was a thin and flexible grass-like stem (Table 1: Tool A), while the following three probes were short (~10–15 cm long; Fig. 1a), rigid sticks collected by the monkey from among those naturally lying on the rocky substrate at the site (Table 1: Tools B–D). Based on these observations we have defined two novel tool-use behaviours for wild capuchins, nasal probe and toothpick, as follows:

**Table 1:** Frequency of nasal probe (NP) and toothpick (TP) behaviours, with accompanying reactions, by an adult female capuchin monkey in Serra da Capivara National Park

Behaviour	Tool A	Tool B	Tool C	Tool D	Total
Nasal probe (NP)	1	8	4	0	13
NP sneeze^a^	0	11	3	0	14
NP lick	1	3	1	0	5
Toothpick (TP)	0	6	0	1	7
TP lick	0	7	0	1	8

The tools were used sequentially (A–D)

^a^We recorded 21 additional sneezes, but could not directly correlate these with tool use

*Nasal probe*: careful insertion of a thin plant tool into the nostril, usually triggering a sneeze reaction, accompanied by inspection of the probe tool.

*Toothpick*: precise placement of one end of a thin plant tool into the mouth, held in position against a tooth or the gum, followed by rapid side-to-side movement of the tool and its inspection once withdrawn from the mouth.

We recorded a total of 13 nasal probe insertions (Fig. 1b), ten of which were followed by one or two sneezes. The nasal probe insertion was done very carefully, and in each case insertion ceased if a sneeze was triggered. Acácia would then vigorously rub her nose with her hand and arm. Only a small volume of mucus, if any, was discharged with each triggered sneeze. Nasal probing produced an average of just over one sneeze per use, and the capuchin licked the tool afterwards on fewer than half of the 13 occasions (Table 1). Licking was more common during toothpick tool use, which never elicited a sneeze. Tooth picking (Fig. 1c) was performed with a quicker motion, but still with care taken to repeatedly place the tip of the probe at the same location inside the mouth. Acácia appeared to be working at, or rubbing the base of the teeth or her gums on the upper right side of her mouth, beneath her lip. Once the tool was in place, she worked the handheld end of the tool rapidly back and forth. We recorded seven instances of toothpick tool use, all of which involved two of the more robust stick probe tools (Tools B and D) rather than the flexible grass tool. Acácia used one of the probes (Tool B) to perform both the nasal probe and toothpick behaviours, interspersing one with the other. She regularly inspected and licked the tools during both types of tool use (Fig. 1d), although we were unable to determine whether this was to remove dislodged material from the tool’s tip or for some other purpose.

## Discussion

The use of a stick tool as a nasal probe and toothpick is idiosyncratic, and may therefore be an innovation on behalf of this female capuchin. Self-directed stick tool use has been noted previously in several wild great ape populations, as well as in wild monkeys (Shumaker et al. 2011). Specific behaviours similar to those described here for the Serra da Capivara capuchin have also occasionally been reported in captive individuals, including at habitual level (McGrew and Tutin 1973; Bayart and Anderson 1985). However, we focus here on wild or free-living animals to avoid potential influences from humans on captive or provisioned primate tool use behaviour (Haslam 2013).

Primate insertion of a stick probe into their own nasal cavity has been reported in detail only for an adult male chimpanzee (*Pan troglodytes schweinfurthii*) in the Mahale Mountains National Park, Tanzania (Nishida and Nakamura 1993; Nishida et al. 2009). Over more than a decade, this chimpanzee used multiple vegetation probes to trigger a sneeze reaction and clear its nasal passage, including while it displayed flu-like symptoms. One other member of the same group at Mahale, an adolescent female, has been observed independently performing the same behaviour, which it did twice in the space of a few minutes (Marchant and McGrew 1999). This behaviour is classified as rare in surveys of putative chimpanzee cultural variants (Whiten et al. 1999, 2001).

While the observed capuchin behaviour did not produce a noticeable volume of mucus, and the individual did not display any flu-like symptoms, we cannot rule out nasal clearing as a potential proximate cause. It may also be that an irritant was lodged in the capuchin’s nasal passage, and if so, it was not dislodged during the several sneezing episodes that were observed over a 5-min period. Neither can we rule out use of the probe tool as simply a self-inspection or curiosity-driven behaviour. The Mahale nasal probing was reported as a potential parallel behaviour to self-medication in chimpanzees, and an example of deliberate manipulation of an involuntary body response (sneezing) to relieve an unpleasant condition (Nishida and Nakamura 1993). While the direct comparison of chimpanzee and capuchin behaviour can be informative (Visalberghi and McGrew 1997), it is unclear at this point whether there is any convergence between these taxa in the rare reports of nasal probing behaviour. The care with which Acácia introduced the probe to her nose, and the regular continuation of the probe insertion until a sneeze was provoked, suggest that sneezing may have been an intended aim of her tool use behaviour, but we cannot speculate further at present.

Stick tool use for cleaning or picking at teeth has been reported for a slightly wider variety of wild primates, although this behaviour has not been described in detail for any of them and to our knowledge none reach habitual level. For example, an adult male bonobo (*Pan paniscus*) at the Wamba site, Democratic Republic of Congo, was observed using twigs to clean his teeth on two occasions, one of which also involved modifying the tool before use (Ingmanson 1996). Long-tailed macaques (*M. fascicularis*) in Central Java, Indonesia, have also been reported to brush their teeth with twigs (Watanabe et al. 2007), and orangutan (*Pongo abelii*) use of sticks as toothpicks has been noted as a present but rare behaviour at the Ketambe and Suaq Bulimba sites in Sumatra (van Schaik et al. 2008; Meulman and van Schaik 2013). The capuchin’s deliberate and repeated use of a probe tool to target the same tooth or gum area suggests that the purpose was either relieving discomfort or dislodging an irritant.

Aside from the proximate mechanisms inducing the observed tool behaviours, which remain unclear, we can consider the wider context within which the behaviour occurred. Foremost is the fact that adult female capuchins have not previously been reported to use probe tools in the wild. Only 3 % (*n* = 13) of SCNP probe tool use has been seen among female capuchins, all of which were juveniles. This report therefore demonstrates that the observed sexual bias does not result from any impediment to such actions in wild adult females, which is as expected from studies of captive capuchins (Westergaard and Fragaszy 1987). We do note, however, that the female probe tool use was self-directed, whereas foraging probes appear to be used exclusively by males at SCNP. Another difference is that Acácia never modified the tool prior to or during its use, while probe use by males involves modification most of the time (64 %) (Falótico and Ottoni 2014). The length of Acácia’s probe tools falls at the low end of foraging probes observed at SCNP, which have a median of 23.3 cm, an average of 27.94 ± 14.97 cm, and a range of 6.5–98.2 cm (Falótico and Ottoni 2014).

Choquito, the male that was grooming Acácia during her probe use, had very close and repeated opportunities to observe her behaviour, but no other capuchins were paying attention to Acácia during the observed tool use session. Future monitoring of the multiple capuchin groups inhabiting SCNP will enable us to determine whether the observed behaviour can spread via social learning to become a tradition, or remains an idiosyncrasy.

## Electronic supplementary material

Online resource 1: video of adult female bearded capuchin (*Sapajus libidinosus*) nasal probe and toothpick tool use, Serra da Capivara National Park, 28 September 2014 (MPG 59,632 kb)
